# Single-incision laparoscopic cholecystectomy for cholecystolithiasis coinciding with cavernous transformation of the portal vein: report of a case

**DOI:** 10.1186/1471-2482-13-10

**Published:** 2013-04-11

**Authors:** Takuro Shirasu, Yoneei Kawaguchi, Junichiro Tanaka, Yoshiro Kubota, Toshiaki Watanabe

**Affiliations:** 1 Department of Surgery, Kikkoman General Hospital, Chiba, Japan; 2 Division of Vascular Surgery, Department of Surgery, Graduate School of Medicine, The University of Tokyo, Tokyo, Japan; 3 Department of Surgical Oncology, the University of Tokyo, Tokyo, Japan

**Keywords:** Cavernous transformation of the portal vein (CTPV), Single-incision laparoscopic surgery (SILS)

## Abstract

**Background:**

Cavernous transformation of the portal vein (CTPV) is a rare vascular deformity. It is thought to be secondary to extra-hepatic portal vein obstruction, with formation of serpiginous collateral vessels around the extra-hepatic bile duct, and even the gallbladder. Surgery is difficult because the vessels have irregular courses, are somewhat fragile and bleed easily. Single-incision laparoscopic cholecystectomy, an emerging procedure for symptomatic cholecystolithiasis, has limitations especially in anatomically complex cases.

**Case presentation:**

We describe a 44-year-old woman with symptomatic cholecystolithiasis. Computed tomography revealed a series of tortuous collateral veins at the liver hilum, with the extra-hepatic portal vein occluded at the level of the spleno-portal junction. However, the distended vessels were not particularly close to the cystic duct. We performed single-incision laparoscopic surgery (SILS) for cholecystectomy via a trans-umbilical incision. By pulling the cystic duct out along with neighboring cavernous vessels, we were able to secure detachment of the cystic duct from Calot’s triangle and ligation of the cystic artery. Total operating time was 132 minutes and blood loss was 370 grams. The patient was discharged on postoperative day 2 with no perfusion abnormalities in the liver.

**Conclusion:**

We must pay meticulous attention to the area of Calot’s triangle when performing SILS cholecystectomy with CTPV. SILS cholecystectomy might be an option in highly experienced facilities.

## Background

In this era of minimally invasive procedures, single-incision laparoscopic surgery (SILS) has become a popular option for cholecystectomy [1,2]. SILS has been widely applied for elective cholecystectomy, but has limitations. The surgical field is somewhat small and the working space is limited, making operative procedures difficult. Therefore, we must often convert to conventional laparoscopy with multiple ports or open laparotomy in cases with complex anatomical features anatomy or unexpected bleeding. Cavernous transformation of the portal vein (CTPV) is a rare vascular variant, arising from extra-hepatic portal vein obstruction [3]. Tortuous vessels surround the hepatoduodenal ligament, making biliary surgery difficult due to the high risk of bleeding. More CTPV cases may be detected with advancements in radiological preoperative imaging. Herein, we describe a successfully managed case. To our knowledge, this is the first report of SILS cholecystectomy for cholecystolithiasis in a patient with concomitant CTPV.

## Case presentation

**Figure 1:**
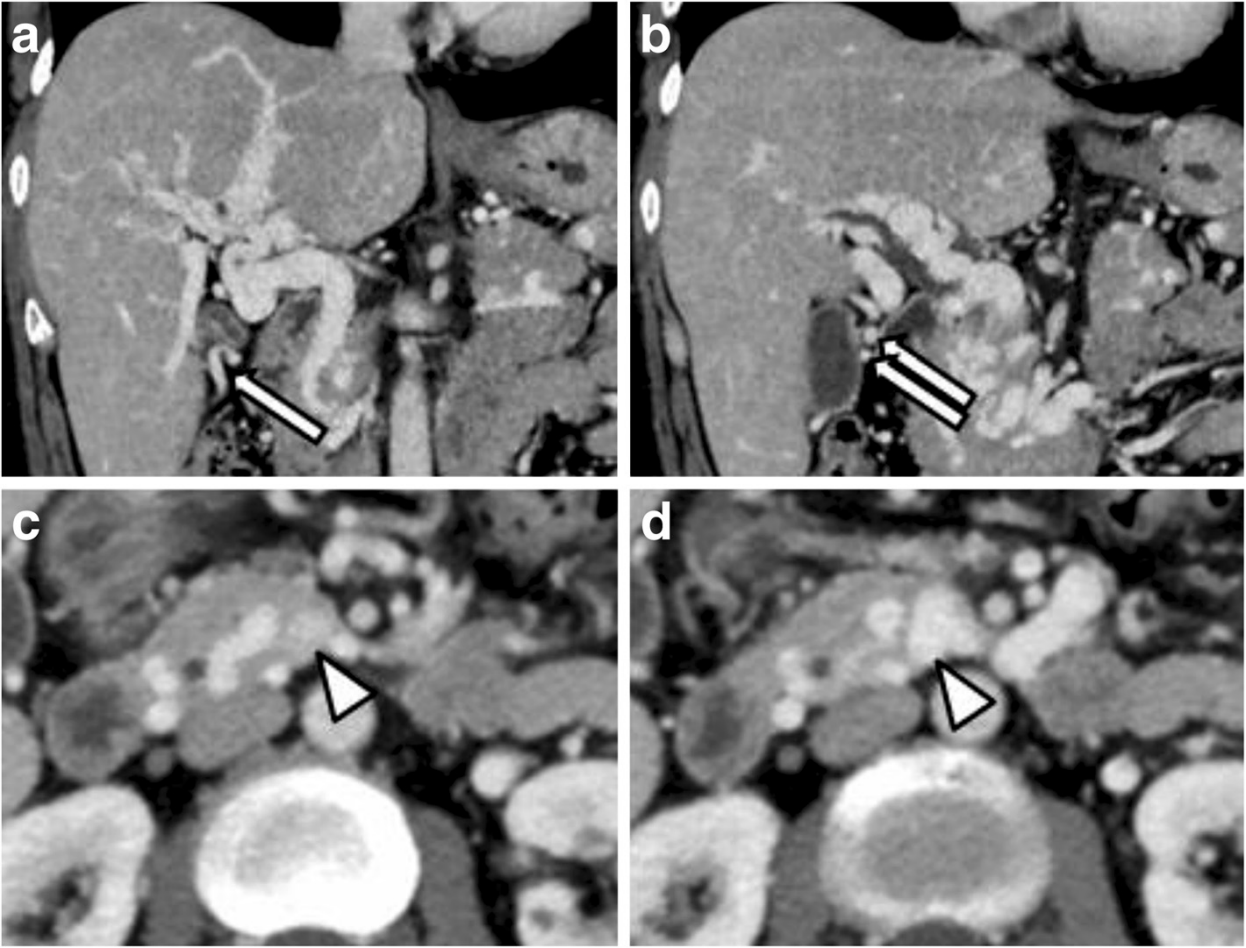
**Arrows show two distended collateral veins, flowing extra-hepatically to the edge of the posterior segment of the liver, both of which were to be sacrificed (a, b).** The extra-hepatic portal vein was occluded near the spleno-portal junction (**c**, **d**).

A 44-year-old Peruvian woman, who had immigrated to Japan 20 years earlier, presented with postprandial upper abdominal pain of four months duration. She was found to have a gallbladder stone, which might have been the cause of her epigastralgia. Her past medical history was unremarkable except for a left renal stone. She had been pregnant three times and given birth to three children. On physical examination, there were no remarkable findings. As to laboratory tests, levels of aspartate amino transferase (AST), alanine amino transferase (ALT), alkaline phosphatase (ALP), glutamyl transferase and total bilirubin were within normal ranges. Ultrasonography showed a few strongly echoic stones but there was no gallbladder wall thickening. Neither intra- nor extra-hepatic bile ducts were dilated. Routine preoperative computed tomography (CT) revealed a series of tortuous collateral veins at the liver hilum, irregularly surrounding the gallbladder from the neck to the fundus (Figure 1a, 1b). The extra-hepatic portal vein was occluded at the level of the spleno-portal junction (Figure 1c, 1d). There were no evidence of hypertrophy of the left liver, splenomegaly or ascites, suggesting that she was not cirrhotic. To check the anatomy of the biliary tract, we performed magnetic resonance imaging, which confirmed discontinuity of the common bile duct. This finding was assumed to have resulted from surrounding collateral vessels rather than a bile duct stone. Our diagnosis was symptomatic cholecystitis with CTPV. After being fully informed of her options, she provided consent to undergo surgery.

We considered SILS to be feasible because the cavernous vessels were not close to Calot’s triangle, and most ran from the middle to the left side of the hepatoduodenal ligament and liver hilum. Two distended collateral veins flowed extra-hepatically to the edge of the posterior segment of the liver. We were determined that these vessels could be sacrificed during the surgical procedure.

**Figure 2:**
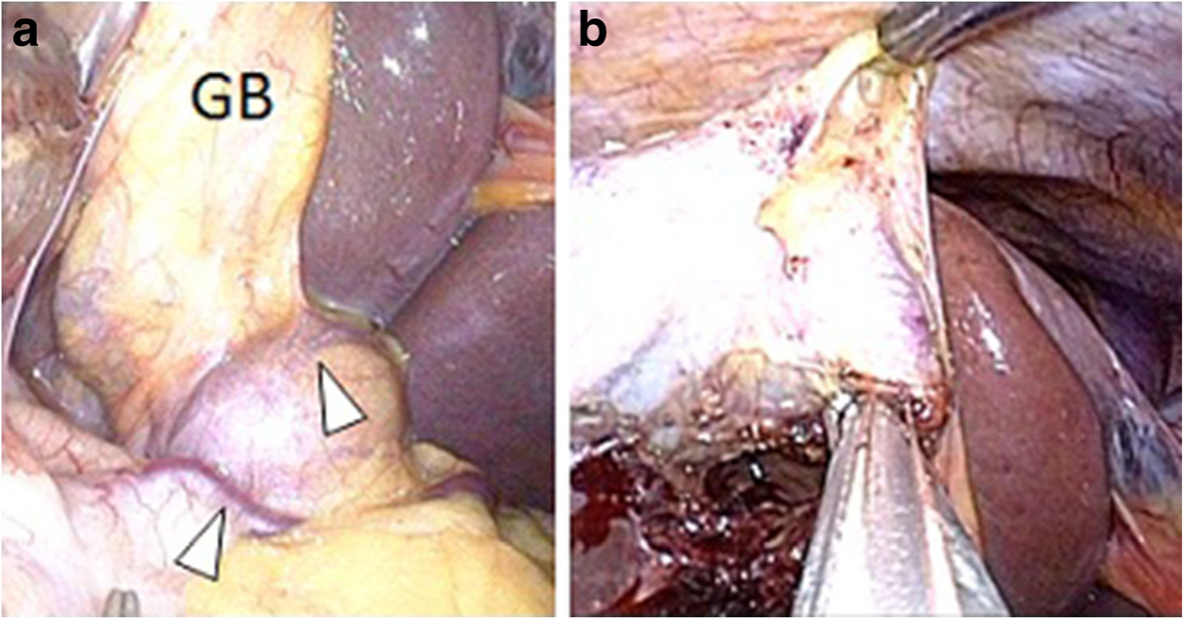
**The courses of cavernous vessels were around the neck of the gallbladder (a, arrowheads).** Detachment of the cystic duct by pulling the cystic duct together with collateral vessels, employing a silk suture (**b**).

The operation was performed under general anesthesia, starting with a trans-umbilical incision. The peritoneal cavity was entered employing the open method, and a LAP PROTECTOR (HAKKO CO.,LTD. NAGANO,JAPAN, TM) was then inserted with EZ ACCESS (HAKKO). There were three holes, for two 5 mm ports and one 12 mm port. A flexible laparoscope (Olympus, Tokyo, Japan, TM) and two operating forceps were used. The pneumoperitoneum was set at 8 mmHg. Suture suspension of the gallbladder was not adopted. The courses of the cavernous vessels surrounded the neck of the gallbladder (Figure 2a). We first approached the cystic duct, with an assistant holding the neck of the gallbladder at the ventral position. Fragile cavernous vessels were encountered in this area, such that we kept the cystic duct together with these vessels. During the entire procedure, this process caused the most bleeding. Pulling the cystic duct out together with collateral vessels, employing a silk suture, we detached the cystic duct and the neck of the gallbladder from Calot’s triangle (Figure 2b). The cystic artery was ligated and transected near the neck of the gallbladder. After confirming the common bile duct, the cystic duct was ligated and clipped. There was bile oozing from the stump of the cystic duct, which was treated by ligation. The remaining surgical procedures were carried out with little difficulty. Hematoma was removed as extensively as possible, and no intra-peritoneal drainage was applied. Total operating time was 132 minutes and blood loss was 370 grams.

The patient was quite well postoperatively, with slightly increased AST (134 U/l), ALT (109 U/l) and ALP (188 U/l) on postoperative day 1, which decreased to 53, 78 and 181, respectively, the next day. Postoperative CT revealed that two extra-hepatic meandering vessels had been sacrificed but there were no hepatic perfusion abnormalities. She was uneventfully discharged on postoperative day 2. The liver enzymes dropped within normal ranges and she was asymptomatic on her last outpatient day of postoperative day 8.

## Discussion and conclusion

With the advent of less invasive operative procedures in every field of surgery, laparoscopic cholecystectomy has replaced open cholecystectomy for cholecystolithiasis. In recent years, SILS cholecystectomy has been widely accepted in numerous facilities [1]. Its potential benefits include superior cosmetic results, reduced postoperative pain and faster recovery with shorter hospital stay. The incidence of intraoperative complications is reportedly 2.7% [1]. A meta-analysis [4] indicated that SILS cholecystectomy takes longer but is as safe as multiport conventional laparoscopic cholecystectomy for uncomplicated cholecystectomy. Our experience with 54 SILS cholecystectomy and 157 conventional multiple-port laparoscopic cholecystectomy (except for converted) cases shows no statistically significant difference in either operative time or blood loss (mean operating time 67.8 min versus 67.1 min, p=0.897 and mean blood loss 6.8 g versus 26g, p=0.103). Furthermore, we have performed SILS in 17 cases and multiple-port laparoscopic cholecystectomy in 18 cases with acute cholecystitis, and the two groups had similar operating times and blood losses (mean operating time 95.8 min versus 81.4 min, p=0.285 and mean blood loss 37g versus 56g, p=0.546).

CTPV was first reported by Gibson et al. [3] in 1955, and is now thought to be secondary to chronic extra-hepatic portal vein obstruction arising from congenital, intra-abdominal inflammatory, traumatic, neoplastic or unknown causes. As Yoshida et al. pointed out [5], an increasing number of asymptomatic cases with CTPV may now be detected with advancements in radiological imaging for preoperative screening. Although there have been few reports examining the influences of CTPV on gallbladder surgery, this condition is a challenge for surgeons because the vessels arise from paracholedocal and pericholedochal venous plexuses, which are somewhat brittle. Takahashi et al. reported a case with asymptomatic CTPV of undetermined cause associated with early gastric cancer [6]. They had difficulty performing lymph node dissection of the hepatoduodenal ligament due to profuse bleeding, amounting to 1500ml. Bockhorn et al. [7] reported surgical outcomes of CTPV with chronic pancreatitis, and noted that greater intraoperative transfusion of red blood cells was required in pancreatic disease patients with CTPV. Surgeons must often surmount the obstacle of bleeding in cases with CTPV.

The long operating time and major blood loss in this case might be points worthy of criticism. We think that the main cause of bleeding is that we tried to detach the cystic duct from neighboring cavernous vessels. These vessels were too fragile to be completely preserved in keeping the cystic duct. The small collateral vessels can be safely sacrificed to minimize bleeding. We believe that laparoscopic cholecystectomy for young women is feasible and that sudden bleeding in this case might have been overcome even if we had performed conventional multiport laparoscopy. A lesson learned from this case is that surgeons performing biliary operations in cases with CTPV might encounter unexpected bleeding. We must be prepared to control intraoperative bleeding regardless of operative procedures.

In conclusion, when we perform SILS cholecystectomy in a case with CTPV, we must pay meticulous attention in the area of Calot’s triangle. SILS cholecystectomy might be an option in highly experienced facilities, but we must always keep in mind that patient safety has the highest priority.

## Consent

Written informed consent was obtained from the patient for publication of this case report and any accompanying images. A copy of the written consent is available for review by the Editor-in-Chief of this journal.

## Abbreviations

ALP: Alkaline phosphatase; ALT: Alanine aminotransferase; AST: Aspartate amino-transferase; CT: Computed tomography; CTPV: Cavernous transformation of the portal vein; SILS: Single-incision laparoscopic surgery.

## Competing interests

The authors declare that they have no competing interests.

## Authors’ contributions

All of the authors were involved in the literature search, writing and final review of this manuscript. All authors read and approved the final manuscript.

## Pre-publication history

The pre-publication history for this paper can be accessed here:

http://www.biomedcentral.com/1471-2482/13/10/prepub
